# IL-22BP controls the progression of liver metastasis in colorectal cancer

**DOI:** 10.3389/fonc.2023.1170502

**Published:** 2023-05-31

**Authors:** Anastasios D. Giannou, Jan Kempski, Tao Zhang, Jöran Lücke, Ahmad Mustafa Shiri, Dimitra E. Zazara, Ioannis Belios, Andres Machicote, Philipp Seeger, Theodora Agalioti, Joseph Tintelnot, Adrian Sagebiel, Miriam Tomczak, Lennart Bauditz, Tanja Bedke, Lorenz Kocheise, Baris Mercanoglu, Mohammad Fard-Aghaie, Emmanouil Giorgakis, Panagis M. Lykoudis, Anastasia Pikouli, Julia-Kristin Grass, Ramez Wahib, Jan Bardenhagen, Benjamin Brunswig, Asmus Heumann, Tarik Ghadban, Anna Duprée, Michael Tachezy, Nathaniel Melling, Petra C. Arck, Pablo Stringa, Maria Virginia Gentilini, Gabriel E. Gondolesi, Ryosuke Nakano, Angus W. Thomson, Daniel Perez, Jun Li, Oliver Mann, Jakob R. Izbicki, Nicola Gagliani, Ioannis C. Maroulis, Samuel Huber

**Affiliations:** ^1^ Section of Molecular Immunology und Gastroenterology, I. Department of Medicine, University Medical Center Hamburg-Eppendorf, Hamburg, Germany; ^2^ Hamburg Center for Translational Immunology (HCTI), University Medical Center Hamburg-Eppendorf, Hamburg, Germany; ^3^ Department of General, Visceral and Thoracic Surgery, University Medical Center Hamburg-Eppendorf, Hamburg, Germany; ^4^ Department of Surgery, University of Patras Medical School, Patras, Greece; ^5^ Mildred Scheel Cancer Career Center HaTriCS4, University Medical Center Hamburg-Eppendorf, Hamburg, Germany; ^6^ Department of Pediatrics, University Medical Center Hamburg-Eppendorf, Hamburg, Germany; ^7^ Laboratory for Experimental Feto-Maternal Medicine, Department of Obstetrics and Fetal Medicine, University Medical Center Hamburg-Eppendorf, Hamburg, Germany; ^8^ ll. Department of Medicine, University Medical Center Hamburg-Eppendorf, Hamburg, Germany; ^9^ Department of Surgery, University of Arkansas for Medical Sciences, Little Rock, AR, United States; ^10^ Division of Transplantation, Department of Surgery, University of Arkansas for Medical Sciences, Little Rock, AR, United States; ^11^ 3rd Department of Surgery, Attiko University Hospital, National and Kapodistrian University of Athens, Athens, Greece; ^12^ Division of Surgery and Interventional Science, University College London (UCL), London, United Kingdom; ^13^ Department General Surgery, Liver, Pancreas and Intestinal Transplantation, Hospital Universitario, Fundacion Favaloro, Buenos Aires, Argentina; ^14^ Instituto de Medicina Traslacional, Trasplante y Bioingeniería (IMETTyB, Concejo Nacional de Investigaciones Científicas y tecnológicas (CONICET), Universidad Favaloro), Laboratorio de Inmunología Asociada al Trasplante, Buenos Aires, Argentina; ^15^ Department of Surgery, Starzl Transplantation Institute, University of Pittsburgh School of Medicine, Pittsburgh, PA, United States; ^16^ Department of Immunology, University of Pittsburgh School of Medicine, Pittsburgh, PA, United States

**Keywords:** metastasis, tumor immunology, IL-22BP, colorectal cancer, cancer therapy

## Abstract

**Background:**

The immune system plays a pivotal role in cancer progression. Interleukin 22 binding protein (IL-22BP), a natural antagonist of the cytokine interleukin 22 (IL-22) has been shown to control the progression of colorectal cancer (CRC). However, the role of IL-22BP in the process of metastasis formation remains unknown.

**Methods:**

We used two different murine *in vivo* metastasis models using the MC38 and LLC cancer cell lines and studied lung and liver metastasis formation after intracaecal or intrasplenic injection of cancer cells. Furthermore, *IL22BP* expression was measured in a clinical cohort of CRC patients and correlated with metastatic tumor stages.

**Results:**

Our data indicate that low levels of IL-22BP are associated with advanced (metastatic) tumor stages in colorectal cancer. Using two different murine *in vivo* models we show that IL-22BP indeed controls the progression of liver but not lung metastasis in mice.

**Conclusions:**

We here demonstrate a crucial role of IL-22BP in controlling metastasis progression. Thus, IL-22 might represent a future therapeutic target against the progression of metastatic CRC.

## 1 Introduction

An increasing number of studies highlight the role of the immune system and the tumor microenvironment in cancer progression (1–4). Notably, immune-checkpoint inhibitors, which aim to activate the anti-tumor immune response, are among the most promising new cancer therapies. However, specific immune responses in chronic inflammation can also promote tumor development. Understanding the mechanisms that control the interaction between the immune system and cancer cells is pivotal for the generation of future, more effective immunotherapies.

The pro-tumorigenic effects of the cytokine interleukin 22 (IL-22) have gained increasing attention in recent years. Although IL-22 can have many beneficial functions such as promoting intestinal integrity, supporting the mucosal barrier function and protecting against genotoxic stress (5–9), it can also act on cancer cells directly and fuel cancer progression (7, 10–14). Those dichotomic effects of IL-22 highlight the importance of a tight control of its activity. Interestingly, IL-22 activity is controlled by another protein called IL-22 binding protein (IL-22BP, IL22RA2), which binds IL-22 and prevents it from signaling through its IL-22RA1 – IL-10R2 heterodimeric receptor (15, 16).

The primary cellular sources of IL-22BP can be different depending on the underlying disease. In the case of inflammatory bowel disease (IBD), CD4+ T cells, dendritic cells and eosinophils have been identified as the primary sources of IL-22BP (17, 18). In contrast, dendritic cells (DCs) are the major source of IL-22BP in the healthy intestine and in colorectal cancer (CRC) (11, 19–21). The regulation of IL-22BP expression is still incompletely understood. While TNFα has been shown to regulate IL-22BP in CD4+ T cells in chronic intestinal inflammation, this cytokine does not regulate IL-22BP expression in DCs (18). One study found that IL-22BP production by monocyte-derived DCs is regulated by retinoic acid (19). Recently, our research group as well as others have shown a crucial role of lymphotoxin signaling in the production of IL-22BP by DCs (20, 21).

IL-22BP has been shown to regulate tumorigenesis in the intestine in mice and humans (11, 20). Indeed, high IL-22BP levels in the primary tumors of patients with colorectal cancer (CRC) are associated with a favorable clinical outcome in terms of the overall survival (20). The presence of distant metastasis is a crucial factor influencing the outcome of patients with CRC. However, it is currently unknown how IL-22BP affects CRC-related metastasis formation and progression. Similarly, the cellular sources and the regulation of IL-22BP in CRC metastasis remain unclear.

Here, we describe a down-regulation of IL-22BP expression in the primary tumors of patients with metastatic CRC compared to patients without lymph node involvement or distant metastasis. In parallel, the expression of IL-22BP is increased in liver metastasis compared to normal liver tissue. Using mouse models, we could show that IL-22BP protects mice from the progression of liver but not lung metastasis. Furthermore, we describe DCs as the primary cellular source of IL-22BP in liver metastasis. Collectively, our data point towards a crucial role of IL-22BP not only in the development of the primary tumor but also in the metastatic process.

## 2 Results

### 2.1 IL-22BP expression is upregulated in liver metastasis

**Figure 1:**
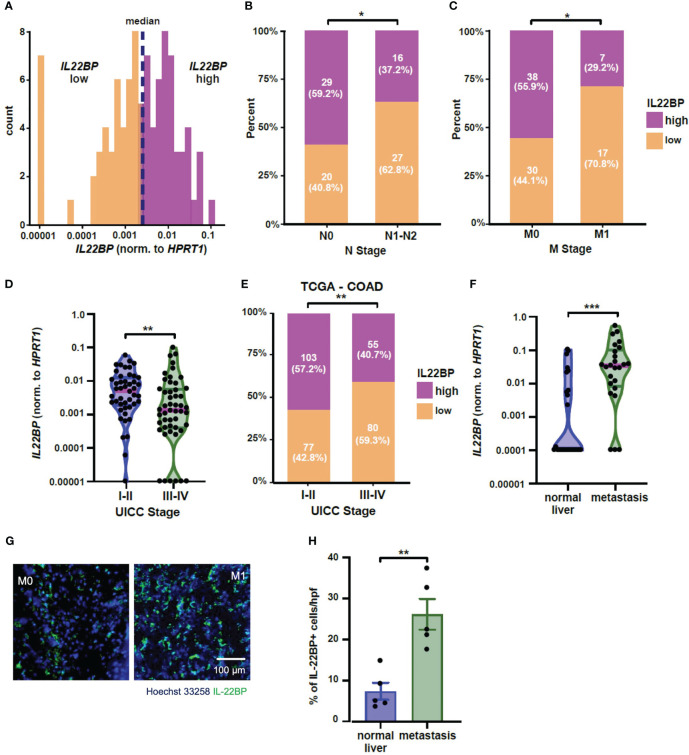
IL-22BP expression is upregulated in liver metastasis. **(A)** Histogram showing the expression levels of *IL22BP* in patients with colorectal cancer and the grouping into high and low *IL22BP* expression using the median value (n=96). **(B)** Bar graph comparing the distribution of high and low *IL22BP* expression in the primary tumor of patients with (N1-N2) or without (N0) lymph node metastasis (n=92). **(C)** Bar graph comparing the distribution of high and low *IL22BP* expression in the primary tumor of patients with (M1) or without (M0) distant metastasis (n=92). **(D)** Violin plot of the expression of *IL22BP* in the primary tumor of patients with early (UICC I-II) or advanced-stage (UICC III-IV) CRC (n=92). **(E)** Bar graph comparing the distribution between high and low *IL22BP* expression and UICC Stage in the publicly available TCGA-COAD dataset. **(F)** Violin plot of the expression of *IL22BP* in the normal liver tissue (n=27) and liver metastasis (n=25). **(G)** Representative immunofluorescence picture showing the presence of IL-22 and IL-22BP in normal liver tissue and liver metastasis. **(H)** The summarizing statistics of the immunofluorescence analysis of IL-22BP+ cells in normal liver tissue and liver metastasis (n = 5). The data presented in **(D)** and **(F)** are the median and interquartile ranges. * = P < 0.05; ** = P<0.01; *** = P < 0.001 as assessed by chi-square test **(B, C, E)** and Mann-Whitney U test (**D**, **F–H**).

We have previously published an association between high IL-22BP levels in the primary tumor and longer survival in patients with colorectal cancer (20). Using the same data set, we again divided the patients based on the expression of *IL-22BP* into patients with high and low expression of this protein (**Figure 1A**). We found a statistically significant relationship between IL-22BP levels and the nodal and metastasis staging of tumors (based on the TNM classification, **Figures 1B, C**). In general, patients with cancers limited to the primary tumor location (i.e., no lymph node involvement or distant metastasis, UICC stage I-II) showed a higher *IL-22BP* expression than patients in which the tumor had already spread to lymph nodes or distant organs (UICC stage III-IV, **Figure 1D**). Importantly, we could confirm this association between UICC tumor stage and IL-22BP expression levels using the publicly available The Cancer Genome Atlas (TCGA) COAD (Colon adenocarcinoma) dataset (**Figure 1E**) (22).

Next, we analyzed the expression of *IL-22BP* in another CRC-patient cohort who underwent resection of their liver metastases. Interestingly, our data showed an upregulation of IL-22BP expression in the liver metastasis compared to the expression in healthy liver parenchyma of those patients (**Figure 1F**). Those findings could be confirmed on a protein level using immunofluorescence (**Figures 1G, H**).

In summary, our data indicate a down-regulation of *IL-22BP* in the primary tumors of patients with advanced-stage CRC. However, the expression of this protein is in parallel increased in the liver metastasis compared to the healthy liver. Based on this data, we hypothesized a crucial role of IL-22BP in the process of CRC metastasis formation.

### 2.2 IL-22BP protects mice from liver metastasis development

**Figure 2:**
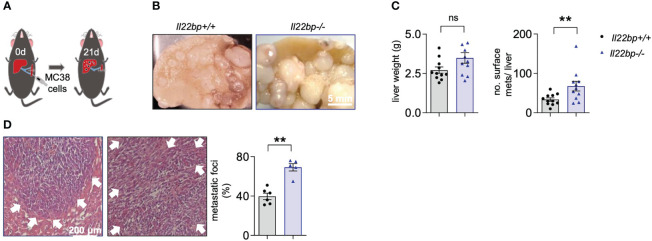
IL-22BP controls the formation of liver metastasis. **(A)** Schematic overview of intrasplenic injection of MC38 cells for forced liver metastasis induction. **(B)** Representative macroscopic pictures of liver metastases in *Il22bp* ^+/+^ and *Il22bp* ^-/-^ mice **(C)** Number of macroscopic liver metastases and liver weight in *Il22bp* ^+/+^ and *Il22bp* ^-/-^ mice. **(D)** Representative microscopic pictures and the corresponding statistics showing the microscopic liver metastases in *Il22bp* ^+/+^ and *Il22bp* ^-/-^ mice as a percentage of the total liver parenchyma. Data presented as mean ± SEM. ** = P<0.01; as assessed by Mann-Whitney U test. ns =p>0.05. White arrows show the boundaries of microscopic liver metastasis.

Our human data suggested a role of IL-22BP in the formation of CRC-related liver metastasis. In the next step, we aimed to test, if IL-22BP plays indeed a causative role in the metastatic process. To this end, we performed an intrasplenic injection of the murine colon carcinoma cell line MC38 in wild type (WT, *Il22bp^+/+^*) and *Il22bp*-deficient (*Il22bp* ^-/-^) mice and compared the generation of liver metastasis 21 days later (**Figure 2A**), as done previously (23, 24). Interestingly, *Il22bp*-deficient mice showed an increased number of macroscopic liver metastases and histologically present metastatic foci (**Figures 2B-D**). Those data indicate a protective effect of IL-22BP on the formation of liver metastasis.

**Figure 3:**
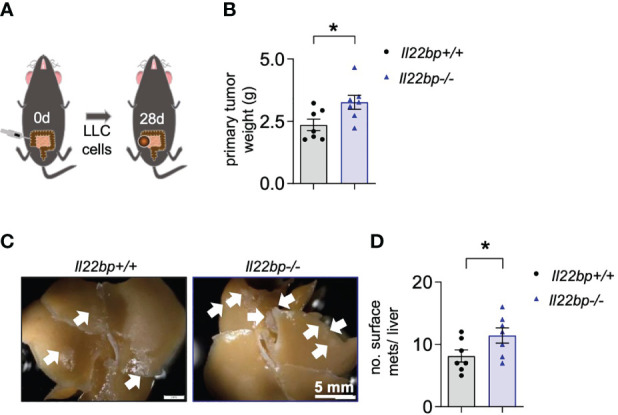
IL-22BP controls the formation of liver metastasis after intracaecal injection of LLC cells. **(A)** Schematic overview of the intracaecal injection of LLC cells for spontaneous liver metastasis induction. **(B)** Primary tumor weight of *Il22bp* ^+/+^ and *Il22bp* ^-/-^ mice 28 days after LLC cell injection **(C)** Representative macroscopic pictures of liver metastases in *Il22bp* ^+/+^ and *Il22bp* ^-/-^ mice. **(D)** Number of liver metastases in *Il22bp* ^+/+^ and *Il22bp* ^-/-^ mice. Data presented as mean ± SEM. * = p≤0.05 as assessed by Mann-Whitney U test. Arrows show the macroscopic liver metastases.

In order to verify those results in another setting, we employed the model of intracaecal injection of the Lewis Lung Carcinoma (LLC) cell line and liver metastasis formation was assessed 28 days after the injection (**Figure 3A**). Indeed, 28 days after the intracaecal injection of the tumor cells, the liver weight and the number of liver metastases was increased in *Il22bp* ^-/-^ mice compared to wild type (*Il22bp^+/+^*) mice (**Figures 3B-D**). Thus, these results confirm that IL-22BP controls the formation of liver metastasis in mice.

### 2.3 IL-22BP does not control formation of lung metastasis

**Figure 4:**
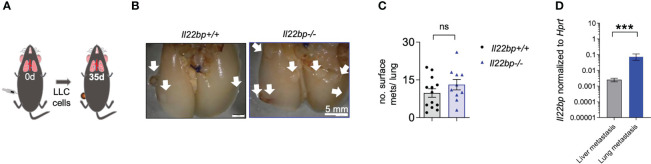
IL-22BP does not influence lung metastasis formation. **(A)** Schematic overview of flank injection of LLC cells for spontaneous lung metastasis induction. **(B)** Representative macroscopic pictures of lung metastases in *Il22bp* ^+/+^ and *Il22bp* ^-/-^ mice. **(C)** Number of lung metastases in *Il22bp* ^+/+^ and *Il22bp* ^-/-^ mice. **(D)** *Il22bp* mRNA expression in lung and liver metastasis (n=7). Data presented as mean ± SEM. ns =p>0.05; *** =p<0.001 as assessed by Mann-Whitney U test. White arrows show the macroscopic lung metastases.

Finally, we aimed to test whether this effect is specific for the seeding of cancer cells into the liver or whether it is a broad mechanism which also applies to metastasis formation into other target organs. To this end, we injected LLC cancer cells into the flank of the mice and assessed lung metastasis formation after 35 days (**Figure 4A**). In contrast to the phenotype observed in the liver, the number of lung metastases was not different between *Il22bp^+/+^* and *Il22bp^-/-^* mice (**Figures 4B, C**). Interestingly, the expression level of *Il22bp* was higher in lung metastasis compared to liver metastasis (**Figure 4D**). Taken together, our results indicate that the absence of IL-22BP favors the development of liver but not lung metastasis and that the metastasis-controlling functions of IL-22BP are dependent on the organ-specific microenvironment.

### 2.4 Innate immune cells are the major cellular source of IL-22BP in liver metastasis

**Figure 5:**
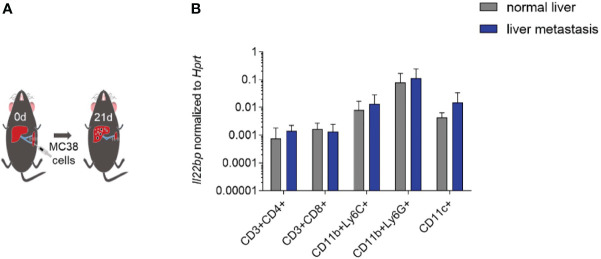
IL-22BP is produced by DCs in liver metastasis. **(A)** Schematic overview of intraplenic injection of MC38 cells for forced liver metastasis induction. **(B)** *Il22bp* expression in CD4^+^ T cells, CD8^+^ T cells, CD11c+ cells, CD11b+ Ly6C+ cells and CD11b+Ly6G+ cells. Results of 2 experiments; in each experiment sorted cells were pooled from 5 mice in each group.

Different cell types have been reported to produce IL-22BP. Both CD4+ T cells and eosinophils have been described as relevant sources of IL-22BP in the inflamed colon of patients with IBD (17, 18). In contrast, DCs are the major source of IL-22BP in the healthy intestine and in CRC (11, 19, 20). To identify the cellular source of IL-22BP, we intrasplenically injected wild-type mice with MC38 cancer cells (**Figure 5A**). After 21 days, the mice were sacrificed and we isolated leukocytes from livers with metastasis and from healthy livers of control mice that were not injected with MC38 cancer cells (and thus did not develop liver metastasis). *Il22bp* expression was analyzed after cellular sorting of T cells, DCs, CD11b+Ly6C+ and CD11b+Ly6G+ cells. Our results demonstrate an increased mRNA expression of *Il22bp* in DCs in liver metastasis. In contrast, the mRNA expression of *Il22bp* did not change in the case of CD4+ T cells, CD8+ T cells, CD11b+Ly6C+ and CD11b+Ly6G+ cells. In general, CD11b+Ly6G+, DCs and CD11b+Ly6C cells produced the highest levels of IL-22BP in liver metastasis (**Figure 5B**). We thus conclude that DCs are not only the major cellular source of IL-22BP in primary CRC tumors, but also are one of the major cellular sources of IL-22BP in CRC-derived liver metastasis.

### 2.5 IL-22BP controls the proliferation of metastatic cells but not the extravasation process

**Figure 6:**
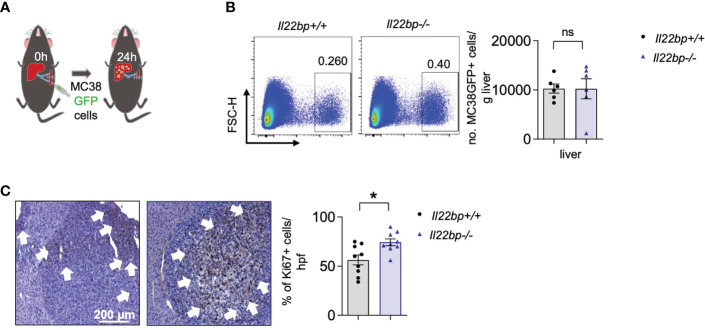
IL-22BP controls the proliferation of metastatic cells but not the extravasation process **(A)** Schematic overview of intrasplenic injection of GFP-labelled MC38 cells for forced liver metastasis induction. **(B)** FACS plots and the corresponding statistics showing the number of GFP+ extravasated MC38 cells in the liver parenchyma of *Il22bp* ^+/+^ and *Il22bp* ^-/-^ mice. **(C)** Representative microscopic pictures and the corresponding statistics of immunohistochemical analysis of Ki-67+ cells in liver metastasis of *Il22bp* ^+/+^ and *Il22bp* ^-/-^ mice. Data presented as mean ± SEM. Data presented as mean ± SEM. ns>0.05; * = p≤0.05 as assessed by Mann-Whitney U test. White arrows point to areas of proliferation rich in Ki-67+ cells.

Metastasis formation is a multi-step process which culminates in the extravasation of circulating tumor cells into the target organ and the subsequent development of a metastatic niche. The proliferation of the metastatic cells then leads to the formation of established metastases in the target organ. We have recently shown that IL-22 plays a crucial role in the extravasation of cancer cells into the liver parenchyma (25). In order to test if IL-22BP plays also a role in cancer cell extravasation into the liver, we performed an *in vivo* extravasation experiment. Specifically, we injected GFP-labelled MC38 cells intrasplenically and counted the number of cells extravasating into the liver after 24 hours using flow cytometry (**Figure 6A**). Notably, we found no difference in the number of extravasating cancer cells between wild type and *Il22bp*-deficient mice (**Figure 6B**). This could be explained by the physiologically very low levels of IL-22BP in steady state conditions in the liver. Indeed, our human data have shown that IL-22BP gets upregulated in already established macro-metastases.

To test if IL-22BP controls the proliferation of metastatic cells that have already extravasated into the liver, we performed a Ki-67 staining of livers isolated from in the injected wild type and *Il22bp*-deficient mice. Interestingly, we found an increased percentage of proliferating tumor cells in *Il22bp*-deficient mice compared to wild type mice (**Figure 6C**). Overall, our data indicate that IL-22BP does not control the early process of cancer cell extravasation into the liver but rather controls the proliferation of tumor cells in already established liver metastasis.

## 3 Discussion

Tumor development is not only influenced by intrinsic genetic changes in the cancer cells but also by the immunological tumor microenvironment (1, 2, 26–28). One factor that has been identified to play a crucial role in the progression of colorectal cancer is IL-22. IL-22 expression is upregulated in CRC and is associated with chemotherapy resistance (10, 11, 13, 29). Notably, the activity of IL-22 is regulated by IL-22BP, a high-affinity, soluble IL-22 receptor that prevents IL-22 from binding to its membrane-bound IL-22 receptor (IL-22RA1) (15, 16). We have recently shown a crucial role of IL-22BP in the development of primary tumors in the intestine in mice and humans (20). In the current study, we examined whether IL-22BP regulates the metastatic cascade in CRC since the prognosis of patients with colorectal cancer is largely dependent on the presence of metastasis. However, due to limited understanding of the mechanisms underlying the metastatic cascade, there are no therapies available that specifically inhibit metastasis formation or progression.

Interestingly, we found that IL-22BP is downregulated in the primary tumors of advanced-stage CRC. Of note, our study uses a relatively small sample size of only 96 patients with CRC for cytokine expression analysis, which may not be representative of the larger population. We have thus extended our study by analyzing the COAD Dataset of The Cancer Genome Atlas (TCGA) (22). Specifically, we compared whether patients with high and low IL-22BP Expression are differentially distributed between patients with UICC Stage I-II (i.e. tumor growth limited to the primary tumor side and UICC Stage III-IV (i.e. dissemination into locoregional lymph nodes or distant organs). Indeed, we could confirm that lymph node involvement and distant metastasis are associated with low IL-22BP expression. Importantly, the findings presented here are limited to patients with CRC and liver metastasis and cannot me generalized to other populations (such as CRC patients with distant metastasis affecting other organs). Finally, it is worth noting that despite the age and gender being equally distributed in both UICC Stage I-II and UICC Stage III-IV patients in our CRC cohort (**Table 1**), other cofounding factors not analyzed in the current study (such as comorbidities) could also potentially affect the expression levels of cytokines.

**Table 1:** Characteristics of Patients with UICC Stage I-II and UICC Stage III-IV CRC.

	UICC I-II	UICC III-IV
Age (± SD)	67.2 (± 10.5)	65.6 (± 11.3)
Sex	Female N = 20	Female N = 18
	Male N = 26	Male N = 28

In contrast to the primary tumor, we found that the expression of IL-22BP is upregulated in liver metastasis compared to healthy liver tissue. Using *IL-22BP* ^-/-^ mice we could show that IL-22BP protects mice from liver metastasis, while it has no effect on the development of lung metastasis. Interestingly, the expression levels of IL-22BP are higher in lung metastasis compared to liver metastasis. Overall, those results suggest that the organ-specific microenvironment might influence the significance and function of IL-22BP in controlling the metastatic cascade.

Metastasis formation is a multi-step process. In the last and crucial steps, circulating tumor cells derived from the primary tumor need to extravasate into the target organ and establish a metastatic niche that promotes the progression of early metastasis. We have recently shown that IL-22 plays a crucial role in mediating the extravasation of cancer cells into the liver (25). IL-22BP does not seem to have such a role, since we here demonstrate that it does not affect cancer cell extravasation into the liver. This may be explained by the fact that the IL-22BP levels in the liver are very low under physiological conditions. However, IL-22BP is strongly upregulated in established liver metastasis. In line with this data, a genetic knock-out of IL-22BP resulted in faster growth of liver metastasis by increasing the proliferation of cancer cells. Thus, we conclude that IL-22BP is a crucial anti-tumorigenic factor that slows down the progression of liver metastasis. Interestingly, these effects are liver-specific since the knock-out of IL-22BP did not influence the formation of lung metastasis.

Dendritic cells, eosinophils and CD4+ T cells have been found to produce IL-22BP. However, the primary source of IL-22BP can differ depending on the disease-specific micro-milieu. Here, we show that CD11b+Ly6G+, CD11c^+^ and CD11b+Ly6C+ cells are the major source of IL-22BP in liver metastasis. However, of those cells only CD11c+ cells appear to upregulate IL-22BP in the setting of liver metastasis. Thus, dendritic cells are not only the major cellular source in the primary tumor in CRC, but they also represent one of the major cellular sources in CRC liver metastasis. Further studies are needed to decipher the mechanism underlying the upregulation of IL-22BP during the formation of liver metastasis. Moreover, the fact that IL-22BP was measured only on mRNA level in our animal experiments represents a limitation of our study. Currently, an antibody-based staining procedure of murine IL-22BP is complex and has not been successfully reported in a consistent way. Overcoming that limitation in future studies could provide further evidence regarding the differential levels of IL-22BP in tumor infiltrating lymphocytes compared to immune cells in the peritumoral tissue. Moreover, the spatial distribution of those cells in the tumor microenvironment could potentially offer explanations for the fact that IL-22BP controls the development of liver metastasis but not lung metastasis.

In summary, our data indicate a crucial role of IL-22BP in the progression of liver metastasis. Since IL-22BP is an endogenous antagonist of IL-22 signaling, our data indicate that pharmacological targeting of IL-22 or via boosting of IL-22BP might represent promising treatment options for CRC patients with liver metastasis. However, further studies are needed to assess how current pharmacological therapies are affecting the IL-22 - IL-22BP axis. Indeed, immunotherapies increasingly effective in various malignancies and are also used now in CRC. Similarly, other chemotherapies such as capecitabine can also affect the immunological response (30). Thus, further research is needed to understand possible synergistic effects of available therapeutic strategies and a pharmacological IL-22 blockade.

## 4 Materials and methods

### 4.1 Animals

*IL-22BP* ^-/-^ mice have been described elsewhere (11). Age- and sex-matched knock-out mice and co-housed in-house bred C57/BL6 WT mice 8 to 18 weeks of age were used for all experiments. All animals were cared for in accordance with the institutional review board ‘Behörde für Soziales, Familie, Gesundheit und Verbraucherschutz’ Hamburg, Germany.

### 4.2 Human cohort used for cytokine expression analysis in primary CRC tumor

Human tissues were obtained freshly after surgical removal of tumors from patients diagnosed with CRC. Both the healthy colonic mucosa and parts of the tumor were collected and frozen until the RNA isolation procedure. In this study, we analyzed the expression levels of cytokines in 96 patients with CRC who were operated between 2010 and 2015 and who did not receive oncological therapy prior to surgical resection. The pathological tumor staging was available for 92 of those patients, the remaining four patients have thus been excluded from the analysis of clinical data. All human studies were approved by the local ethical committee (Ethik-Kommission der Ärztekammer Hamburg).

### 4.3 Human cohort used for cytokine expression analysis in liver metastasis

Human samples obtained from patients with suspicion of Non-alcoholic fatty liver disease, who showed normal histological findings and liver metastasis from CRC patients.

### 4.4 Immunofluorescence

In brief, human liver or liver metastasis slides of tissue were fixed for 10** min** in 4% PFA at RT. Tissues were washed with PBS and incubated in PBS-Triton 0.3% for 5** min**. After washing they were incubated for 60** min** in blocking buffer. Samples were stained overnight with α-IL-22BP antibody (primary mouse anti-mouse antibody, 1:350, antibody from R&D Systems, MAB 10871; Minneapolis, MN). After washing primary antibody (Alexa Fluor 488 rabbit anti-mouse IgG, Invitrogen) staining was performed (1 hour, RT) followed by 5** min** staining with Hoechst 33258 (1:5000). For isotype control, the primary antibody was omitted.

### 4.5 Intrasplenic (i.s.) cancer cell injection for forced liver metastasis induction

For induction of forced liver metastasis, mice received 250 μl PBS containing 3x10^5^ MC38 cells i.s. The injection was performed in the hemi-spleen which was removed 3** min** after cancer cell injection. The mice were sacrificed after 3 weeks. Liver macroscopic metastases were counted by using a stereoscope (Olympus Corporation, Germany) (23, 24).

### 4.6 Flank model for spontaneous lung metastasis induction

For induction of solid tumors and subsequent spontaneous lung metastasis formation, mice were anesthetized using isoflurane inhalation and received a 100 μl PBS containing 5x10^5^ LLC cells subcutaneously (s.c.) (24). The tumors were resected after 2 weeks. The mice were sacrificed 4 weeks after tumor resection and lung macroscopic metastases were counted by using a stereoscope.

### 4.7 Caecum model for spontaneous liver metastasis induction

The caecum of anesthetized mice was exteriorized through an abdominal laparotomy. 1×10^6^ LLC cells were injected into the caecal wall between the mucosa and the muscularis externa layers using a 30-gauge needle. A proper implantation into the caecum was confirmed at day 0 by a localized bubble in the cecal wall. The mice were sacrificed after 4 weeks and liver macroscopic metastases were counted by using a stereoscope (31).

### 4.8 Hematoxylin and eosin (H&E) staining

Liver specimens were fixed in 4% buffered formalin, and embedded in paraffin or OCT (Sakura, Tokyo, Japan) and stored at -80°C. Tissue sections (4 μm) were prepared and stained with H&E. Metastatic lesion areas were quantified by Image J (ImageJ, U.S. National Institutes of Health, Bethesda, MD).

### 4.9 Quantification of liver metastasis using histological sections

Paraffin liver sections from *Il22bp^+/+^* and *Il22bp^-/-^* mice were stained with hematoxylin and eosin. Images were obtained at a 20x magnification and analysis was conducted using NIH Image J software. For estimation of metastatic site surface (as percentage of liver surface), a point counting grid (7 horizontal and 12 vertical lines/84 points) was superimposed on the images of liver parenchyma fields. The volume fraction of the metastatic foci was then equal to Pi/Pt, where Pi indicates the number of points on the structure of interest, namely on metastatic sites, and Pt is the total number of points on the reference field, which in this case is the liver parenchyma. Therefore, the volume fraction of metastatic sites was expressed as a percentage of the whole liver parenchyma.

### 4.10 Fluorescence activated cell sorting

Fc-γ receptors were blocked using a mAb (clone 2.4G2). The cells were stained with fluorochrome-conjugated antibodies as described elsewhere (25). BD LSRFortessa and FACSAria (BD Biosciences, San Jose, CA) were used for cell analysis and cell sorting, respectively. Data were analyzed using FlowJo v.6.1 (TreeStar, Ashland, OR).

### 4.11 RNA expression analysis

Total RNA was extracted from colon tissue and cells from colon, lymph nodes, liver and spleen using TRIzol^®^ Reagent (Invitrogen). The High capacity cDNA synthesis Kit (Applied Biosystems) was used for cDNA synthesis. Primers and probes were purchased from Applied Biosystems. For mouse *Il22bp* (*Il22ra2*) expression the Mm01192969_m1 primers (Applied Biosystems) were used; for human *IL-22BP* (*IL22RA2*) expression the Hs00364814_m1 primers (Applied Biosystems) were used. Real-time PCR was performed using the Kapa Probe Fast qPCR Master Mix (Kapa Biosystems) on the StepOne Plus system (Applied Biosystems). For both human and mouse, relative expression was normalized to HPRT and calculated using the 2^-ΔΔCt^ method.

### 4.12 Extravasation assay

For the ***in vivo*** extravasation assay, mice received 250 μL PBS containing 3x10^5^ MC38 GFP-labelled cells i.s. The mice were sacrificed after 24h and their livers were weighed. To isolate the MC38 GFP-labelled cells, the murine livers were cut in small pieces and minced using a scalpel. The tissues were incubated for 30** min** at 37°C on a shaking incubator in HBSS (with Ca^2+^ and Mg^2+^) with Collagenase (1 mg/ml) and DNase I (10 U/ml) and supernatants were collected. Then, the supernatants were centrifuged for 4** min** at 40g. We repeated this step twice more. Subsequently, the hepatocytes were removed and the cells were diluted in PBS 1x. One fifth of the cells were mixed with beads in 1:10 dilution and were analyzed by flow cytometry. In every extravasation assay, we used a mouse injected with not labelled cells in order to set up the gate of GFP+ cells.

### 4.13 Statistical analysis

Sample size was calculated using G*power (http://www.gpower.hhu.de/) assuming α = 0.05, β = 0.8, and ρ = 0.3. Statistical analysis was performed with GraphPad Prism^®^ Software (GraphPad Software, San Diego, CA, USA). For comparison of groups, the non-parametric two- sided Mann–Whitney test or Fisher’s exact test was used. The significance level a was set to 0.05.

## Data availability statement

The original contributions presented in the study are included in the article/supplementary material. Further inquiries can be directed to the corresponding authors.

## Ethics statement

The studies involving human participants were reviewed and approved by Ärztekammer Hamburg. The patients/participants provided their written informed consent to participate in this study. The animal study was reviewed and approved by Behörde für Justiz und Verbraucherschutz, Lebensmittelsicherheit und Veterinärwesen (Hamburg, Germany).

## Author contributions

AG and JK collaboratively conceived, designed and carried out most of the experiments, analyzed the data, provided critical intellectual input, and wrote the paper draft. TZ, JLu, AMS, DZ, IB, AM, PSe, TA, JT, AS, MTo, LB, TB, LK, BM, MF-A, AP, J-KG, RW, JB, BB, AH, TG, AD, MTa, and NM performed experiments. EG, PL, PA, PSt, MG, GG, RN, AT, DP, JLi, OM, JI, and NG provided critical intellectual input. IM and SH collaboratively conceived and designed most experiments, supervised the study, and provided critical intellectual input for the final paper draft. All authors contributed to the article and approved the submitted version.
